# Next-Generation Sequencing in Congenital Eye Malformations: Identification of Genetic Causes and Comparison of Different Panel-Based Diagnostic Strategies

**DOI:** 10.3390/ijms26209854

**Published:** 2025-10-10

**Authors:** Lukas Neuhann, Andreas Laner, Elke Holinski-Feder, Teresa Neuhann

**Affiliations:** 1 Department of Ophthalmology, LMU University Hospital, LMU Munich, 80336 Munich, Germany; 2 Medizinisch Genetisches Zentrum (MGZ) München, 80335 Munich, Germany

**Keywords:** congenital eye malformations, microphthalmia-anophthalmia-coloboma, anterior segment dysgenesis

## Abstract

Congenital eye malformations like microphthalmia–anophthalmia–coloboma (MAC), anterior segment dysgenesis (ASD), primary congenital glaucoma (PCG) and congenital cataracts (CC) are significant causes of childhood visual impairment. Phenotypic heterogeneity often complicates diagnosis. The goal of this study was to optimize the diagnostic strategy for next-generation sequencing (NGS)-based procedures, thereby aiming to identify genetic causes of congenital eye malformations. Forty patients with congenital eye malformations were included. A primary diagnostic testing (PD) of a limited number of genes was followed by multigene panel (MGP) testing, including 186 eye-related genes, and exome sequencing. Causative variants were identified in 17 patients (43%) and clinically relevant variants of uncertain significance (VUS) in 6 patients (15%). PD had a diagnostic yield (DY) of 15%, MGP of 29% and exome sequencing of 4%, leading to a cumulative DY of 43%. Diagnostic rates were highest in CC (75%), bilateral cases (46%), complex ocular phenotypes (78%), patients with extraocular manifestations (55%) and positive family history (70%). Rare and possible new genotype–phenotype correlations and candidate genes (*FAT1*, *POGZ*) could be identified. In total, eight (likely) pathogenic variants in six genes (*CYP1B1*, *ADAMTS18*, *MAB21L2*, *NHS*, *MFRP*, *CRYBB1*) were not yet reported. A stepwise genetic testing approach starting with a broad multigene panel followed by exome sequencing provides higher diagnostic yield than limited phenotype-specific testing. Comprehensive genetic diagnosis is essential for prognosis, treatment and genetic counseling, underscoring the need for routine genetic testing and interdisciplinary collaboration in managing congenital eye malformations.

## 1. Introduction

Congenital eye malformations pose significant diagnostic and therapeutic challenges and are a major cause of early-onset visual impairment, possibly impacting normal childhood development. The most common malformations include the microphthalmia–anophthalmia–coloboma (MAC) spectrum, anterior segment dysgenesis (ASD), primary congenital glaucoma (PCG) and congenital cataracts (CC). Although individually rare (e.g., MAC: 0.3–0.66 per 10,000 [1]; CC: 3.5–15 per 10,000 [2,3]), these anomalies collectively account for a substantial proportion of childhood blindness globally [4].

The etiology is highly heterogeneous [3,4]. While environmental factors (e.g., intrauterine infections) may be involved in isolated cases, most malformations are genetically driven [5]. They may present isolated or as first manifestation of complex syndromes involving extraocular manifestations [2,6]. Over 100 genes have been associated with congenital ocular anomalies, but in around 50% of cases, no genetic cause is identified despite extensive testing [6].

An accurate molecular diagnosis is essential for prognosis, recurrence risk assessment [2], targeted surveillance (e.g., stroke risk in *COL4A1* [7]) and eligibility for emerging gene therapies (e.g., *RPE65*-associated retinal dystrophy [8]). Prior to next-generation sequencing (NGS), diagnostics relied on phenotype-based single-gene testing, often missing the underlying cause due to overlapping or atypical presentations [2,9]. NGS now allows broader, parallel analysis of multiple genes or exomes, improving diagnostic efficiency [6,10]. However, challenges remain due to high phenotype–genotype heterogeneity and unknown genetic causes [10,11]. This study investigates the effectiveness of a cross-phenotype multigene panel (MGP) and exome sequencing in patients with MAC, ASD/PCG and CC, comparing diagnostic yields (DY) with small phenotype-specific panels routinely applied in clinical practice, and aims to identify novel or rare variants to guide future diagnostic strategies.

## 2. Results

### 2.1. Study Population Characteristics and Sequencing Results

This cohort included 40 individuals with congenital ocular malformations: 22 with MAC, 14 with ASD and/or PCG and 4 with CC. Of these, 77% had a simple ocular phenotype, 23% a complex one; 72% had isolated eye involvement, while 28% showed syndromic features. Most cases (92%) were bilateral. A positive family history was present in 25%, with sequence data available for relatives in half of the cases. The cohort was 63% female and 37% male. For analysis, ASD and PCG were grouped and mixed MAC/CC cases were classified under MAC.

As illustrated in Table 1, across all three diagnostic levels—PD, MGP and exome—causative variants (ACMG class 4 or 5) were identified in 43% (17/40), including two cases where NGS suggested deletions confirmed by Multiplex ligation-dependent probe amplification (MLPA). Six causative variants were not previously described. PD found causative variants in 15% (6/40): four in ASD/PCG, two in CC and none in MAC. Among the 34 unresolved cases, the MGP identified causative variants in 29% (10/34): 36% in MAC (8 cases), 7% in ASD/PCG (1 case) and 25% in CC (1 case). Exome analysis of the remaining 24 unsolved cases revealed one additional novel variant (2.5% overall) in ASD/PCG. No further variants were found in MAC or CC.

### 2.2. Phenotype-Subgroup Results

#### 2.2.1. MAC

**Figure 1:**
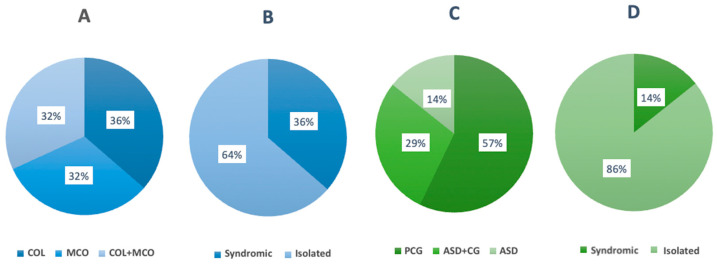
Distribution of clinical features in patients with MAC (**A**,**B**) and ASD/PCG (**C**,**D**). COL = coloboma, MCO = microphthalmia, PCG = primary congenital glaucoma, ASD = anterior segment dysgenesis.

This was the largest clinical subgroup, including 22 patients. Of these, 36% had coloboma, 36% microphthalmia and 28% both (no patient with anophthalmia). Most cases (91%) were bilateral and 23% had additional ocular findings, such as cataracts or persistent hyperplastic primary vitreous. Syndromic features occurred in 36% and 18% had a positive family history (Figure 1A,B). Causative variants (ACMG class 4 or 5) were identified in 36% (8/22), all with bilateral phenotypes, while none were found in unilateral cases. Five genes were involved, all previously associated with MAC. The most common was *MAB21L2* (3 cases) followed by *NHS* (2 cases) and *BCOR*, *MFRP* and *PRSS56* (1 case each). Four variants were novel: *MAB21L2* c.58del (p.Cys20Valfs*37), *NHS* c.320del (p.Gly107Alafs*89) and c.3659dup (p.Asn1220Lysfs*5) and *MFRP* c.1534T>G (p.Cys512Gly).

#### 2.2.2. ASD/PCG

This subgroup included 14 patients. Eight (57%) had isolated PCG, six (43%) had various forms of ASD, four of which (67%) developed glaucoma in early childhood. Two patients had ASD without glaucoma. Other ocular abnormalities, such as microcornea, high myopia and corneal changes, were noted in two patients (14%) and two patients had extraocular features (Figure 1C,D). Thirteen patients (93%) had bilateral disease and four (29%) had a positive family history. Causative variants (ACMG class 4 or 5) were identified in six patients (43%), all in bilateral cases and none in unilateral cases. Four genes, all previously associated with ASD/PCG, were involved: *FOXC1* (two cases), *CYP1B1* (two cases), *PITX2* (one case) and *ADAMTS18* (one case). Two variants were novel: *ADAMTS18* c.1399G>T (p.Gly467*) and *CYP1B1* c.1076_1082dup (p.Asp361Glufs*16). In patient 22, a deletion of the 3‘-region of exon 1 in *FOXC1* was confirmed by MLPA. This specific deletion had not been previously reported.

#### 2.2.3. CC

All four recruited patients with CC had bilateral involvement. Two also had microcornea and one had extraocular abnormalities. Two patients had a positive family history. Causative variants (ACMG class 4 or 5) were found in three of four patients (75%) in *COL4A1*, *PAX6* and *GCNT2*—all genes known to be associated with CC and all previously described in the literature.

### 2.3. Diagnostic Yield

Diagnostic yield (DY) refers to the proportion of patients in which a causative genetic variant (ACMG class 4 or 5) was identified. It varied significantly by diagnostic approach and phenotype.

**Figure 2:**
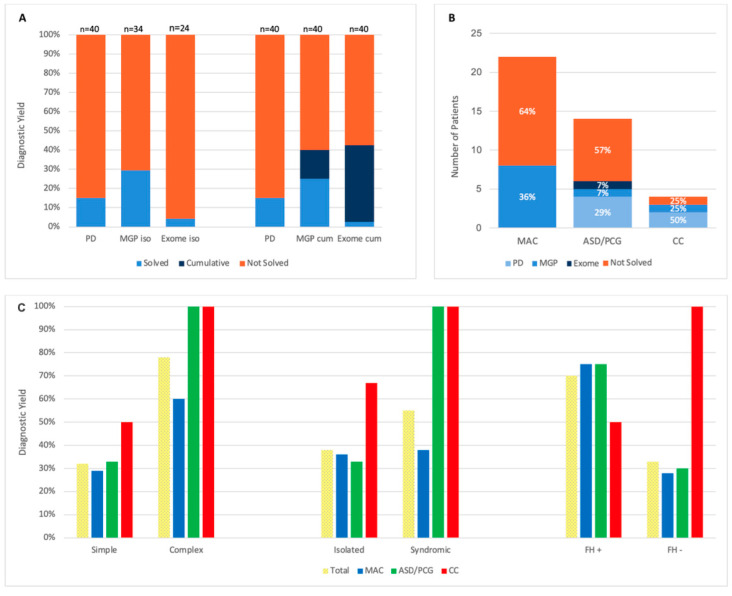
Diagnostic yield—(**A**) for each diagnostic step, (**B**) by phenotypic groups and (**C**) by different clinical feature subgroups. PD = primary diagnostics, MGP = multigene panel, n = number of patients, iso = isolated, cum = cumulative, FH +/− = family history positive/negative.

PD identified causative variants in 6 of 40 patients (15%). The multigene panel (MGP) resolved 10 additional cases, raising the cumulative yield to 40%, as it included all PD genes. Exome sequencing of 24 unresolved cases revealed one more variant (4%), bringing the overall DY to 43% (Figure 2A).

**Figure 3:**
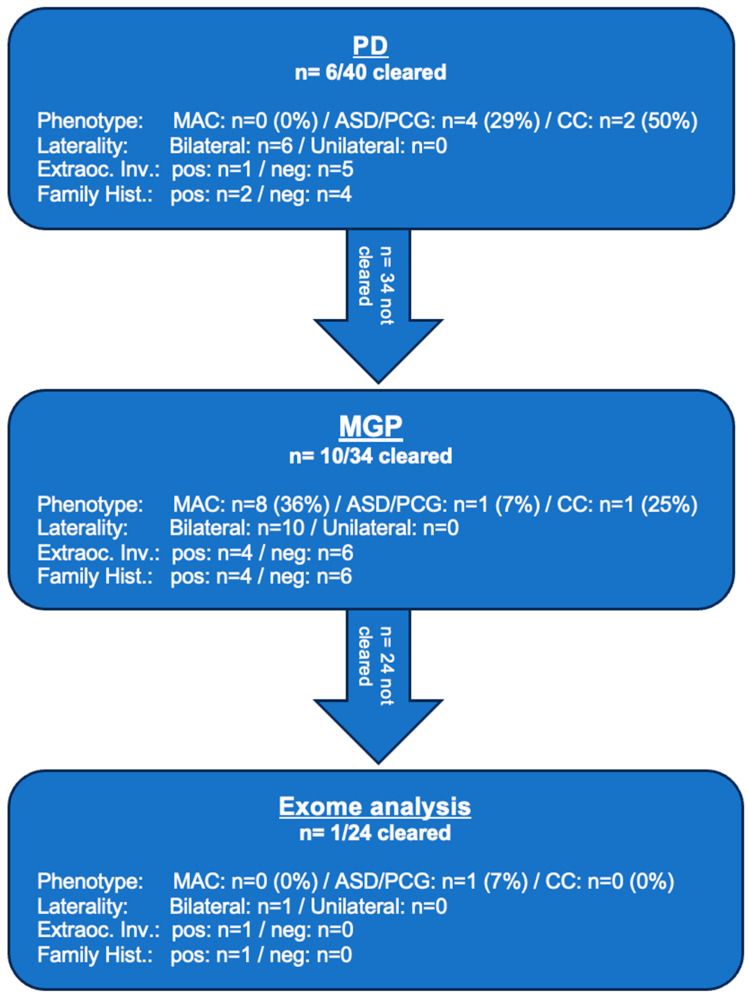
Flowchart of results obtained through each subsequent diagnostic step. Extraoc. Inv.: = extraocular involvement, Family Hist. = Family History, pos = positive, neg = negative.

Among phenotype groups, the highest yield was in CC (75%), followed by ASD/PCG (43%) and MAC (36%) (Figure 2B). No causative variants were found in unilateral cases, while bilateral phenotypes had a 46% yield (CC: 75%, ASD/PCG: 46%, MAC: 40%). Patients with simple ocular phenotypes had a 32% DY (CC: 50%, ASD/PCG: 33%, MAC: 29%) versus 78% in those with additional ocular findings (CC: 100%, MAC: 60%, ASD/PCG: 100%). Syndromic or extraocular features were associated with a 55% yield (CC: 100%, ASD/PCG: 100%, MAC: 38%) compared to 38% (CC: 67%, ASD/PCG: 33%, MAC: 36%) in isolated ocular cases. A positive family history increased DY to 70% (MAC: 75%, ASD/PCG: 75%, CC: 50%), while cases without it had a 33% DY (CC: 100%, ASD/PCG: 30%, MAC: 28%) (Figure 2C). Figure 3 illustrates the results obtained in each diagnostic step.

### 2.4. Variants of Unknown Significance and Candidate Genes

In six patients, variants of uncertain significance (VUS; ACMG class 3) with possible relevance to the observed phenotypes were identified in five genes (*POGZ*, *COL18A1*, *FAT1*, *MFRP*, *CRYBB1*) (Table 2).

**Figure 4:**
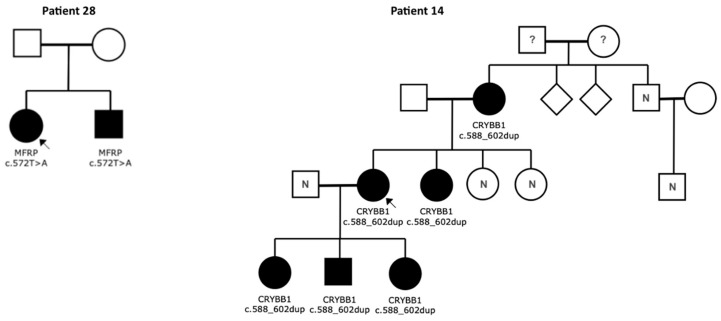
Segregation analysis in patients with Variants of Unknown Significance. ? = phenotype not known, N = variant not present, arrow = index patient.

In patient 14, a novel duplication c.588_602dup (p.Gln197_Tyr201dup) in *CRYBB1* was found in a patient with bilateral cataracts. The variant segregated in all affected family members (Figure 4). Despite its formal classification as VUS, when viewed in the context of clinical and genetic data, this variant is to be considered as highly likely causative, especially since no other causative variant could be identified. Patient 28 with bilateral microphthalmia carries a novel homozygous variant c.572T>A (p.Ile191Lys) in *MFRP*, which currently is classified as VUS. However, this variant is very likely to be pathogenic, according to the clinical and genetic findings, especially since the same variant was confirmed in the similarly affected sibling (Figure 4).

## 3. Discussion

The study aimed to identify genetic causes of congenital eye malformations using extended analysis of NGS data and to compare the DY across different testing strategies. In 17 of 40 patients, pathogenic or likely pathogenic variants (ACMG class 4 or 5) were found. Additionally, two patients carried VUS (ACMG class 3) in *CRYBB1* and *MFRP* that were likely causative based on clinical correlation, raising the DY to 48% overall.

The MGP showed the highest standalone yield and increased the detection rate by 2.5-fold compared to PD, while WES added minimal additional findings.

These results align with previous studies showing detection rates between 24.5% and 75% for broad eye panels [10,22] and support the conclusion that phenotype-spanning MGP and WES significantly outperform limited, phenotype-specific gene panels in detecting genetic causes of congenital eye malformations.

Consistent with findings from other studies [10,23], the DY in this study was highest for CC (100%), followed by ASD/PCG (43%) and MAC (41%), including VUS in *MFRP* (MAC) and *CRYBB1* (CC), which can clinically be considered causative.

No pathogenic variants were found unilateral cases, while the DY in bilateral cases was 46%, consistent with previous research and suggesting that unilateral cases may be more influenced by non-genetic factors, such as intrauterine infection, disruption or undetectable mosaicism [24,25].

DY was higher in cases with additional ocular abnormalities, extraocular or syndromic features and/or positive family history than in isolated or non-syndromic cases and/or negative family history. In ASD/PCG and CC, DY was higher in patients with extraocular features, whereas in MAC, it was comparable regardless of extraocular involvement. This contrasts with previous results suggesting a higher detection rate in isolated CC and in MAC cases with syndromic presentation [6,10].

Overall, the results of this study are largely consistent with existing literature, though differences in study design, patient selection, gene panel composition and variant classification limit direct comparisons. Nevertheless, the data indicate that the highest likelihood of identifying a causative variant occurs in patients with bilateral disease, a positive family history and complex or syndromic ocular phenotypes. In contrast, patients with unilateral involvement and no family history are less likely to receive a molecular diagnosis.

Biallelic variants in *CYP1B1* are the most common cause of autosomal recessive PCG and often linked to corneal opacities presenting as edema, Haab’s striae or buphthalmos [26]. However, patient 27 with *CYP1B1* variants showed a different primary phenotype with diffuse corneal opacity and iris hypoplasia, while glaucoma was only diagnosed secondarily. This matches reports of a rare frosted-glass-like opacity affecting the entire cornea, independent of intraocular pressure [27], expanding the phenotypic spectrum of *CYP1B1*-related disease. In patient 8 with a classical presentation of PCG, a novel frameshift variant c.1076_1082dup (p.Asp361Glufs*16) was identified.

The *ADAMTS18* gene plays an essential role in embryonic eye development and biallelic variants have been linked to MMCAT syndrome (microcornea, myopic chorioretinal atrophy, telecanthus) microcornea and rod–cone dystrophy, early childhood cataracts and/or ectopia lentis and retinal dystrophy without anterior segment features [28]. In patient 20 and the similarly affected sister, exome sequencing revealed a novel homozygous pathogenic variant c.1399G>T (p.Gly467*). The phenotype of the patients included bilateral Axenfeld–Rieger Syndrome (ARS), which has not been previously reported in *ADAMTS18*-related eye disorders, as well as microcornea, high myopia, macular changes, taurodontism and craniofacial anomalies. These findings extend the known clinical spectrum and support the role of *ADAMTS18* in combined anterior and posterior segment malformations.

The *COL4A1* variant c.2317G>A (p.Gly773Arg), previously reported in patients with bilateral CC and neurological features [14], was found in patient 30, who primarily showed a purely ocular phenotype with bilateral CC and microcornea. However, further work-up revealed a past cerebral hemorrhage contributing to a minor motor delay. This highlights the phenotypic variability of *COL4A1*-related disease and underlines the importance of genetic testing in children with CC. Patient 33 carried the missense variant c.113G>A (p.Arg38Gln) in *PAX6*, which is critical for eye and CNS development [29]. While this variant was previously described with bilateral CC and additional features like nystagmus or ASD and microphthalmia [15,29], we report a particularly mild expression with only bilateral CC and microcornea.

*MAB21L2* is involved in ocular and skeletal development and has been linked to MAC spectrum disorders with or without extraocular abnormalities [11,18]. Primarily missense variants are associated with autosomal dominant eye malformations. In this study, the novel heterozygous frameshift variant c.58del (p.Cys20Valfs*37) was found in two unrelated patients with isolated bilateral iris/retina/choroid colobomas, supporting monoallelic loss-of-function variants as a cause of milder, non-syndromic phenotypes. These results expand the phenotypic spectrum of *MAB21L2* and confirm its relevance in isolated ocular malformations without systemic involvement.

*CRYBB1* encodes a lens protein involved in non-syndromic CC [6]. The heterozygous variant c.588_602dup (p.GIn197_Tyr201dup) was identified in patient 14 and five affected family members. Although classified as a VUS, its segregation, conservation and absence in databases suggest likely pathogenicity. This variant is reported here for the first time.

A recent study described a homozygous frameshift variant in *FAT1* in connection with microphthalmia or coloboma and syndromic features in five families and demonstrated its implication in optic cup development in a mouse model [30]. However, a definite gene-disease correlation is not yet established. Patient 26 with bilateral colobomas carried a novel heterozygous variant c.1025G>C (p.Gly342Ala). While its pathogenicity remains uncertain (VUS), bioinformatic analysis suggests a damaging effect. This supports *FAT1* as a promising candidate gene for MAC spectrum disorders.

*POGZ* is linked to White–Sutton syndrome, a rare neurodevelopmental disorder with variable features including ocular anomalies, such as retinal dystrophies; colobomas have only described in isolated (*n* = 3) patients [21,31]. Patients 1 and 5 with *POGZ* variants both presented with coloboma and mild developmental delays. Patient 1 carried a novel missense variant(c.3271C>T (p.His1091Tyr)), while the missense variant found in patient 5 (c.4086A>C (p.Glu1362Asp)) has been previously described as a VUS in a patient with isolated congenital diaphragmatic hernia [21]. Although classified as VUS, bioinformatic analysis and rarity in databases underscore *POGZ* as an interesting candidate gene for congenital eye malformations and the possible association of White–Sutton Syndrome with coloboma.

NGS significantly improves the detection of genetic causes in congenital eye malformations. Cross-phenotype multigene panels (MGP) and exome sequencing consistently outperform phenotype-specific single-gene tests, especially given the high clinical and genetic heterogeneity. This study highlights that rare variants in genes like *MAB21L2*—missed in initial small phenotype-based panels—were the actual cause in several MAC cases, while in former times commonly implicated genes (e.g., *SOX2*, *OTX2*) [32] were not involved. Comprehensive MGP or WES avoid miss-selection of subpanels, save time and are increasingly cost-effective. A stepwise evaluation based on WES offers a practical balance between MGP and WES. While an MGP possibly provides better analysis of the included gene in these specific regions, including alleviation of the assessment of dosage alterations, WES allows broader testing of rare cause including novel associated genes not yet included in panels [22,33]. Therefore, in clinical routine diagnostics it is especially advisable to use a combined approach with primary analysis of an MGP on the base of exome sequencing with possible sequential exome analysis in selected cases. Since no diagnostic approach can detect all causative variants, in specific cases additional genetic methods need to be considered, as was demonstrated in two patients in our cohort where deletions were confirmed by MLPA after this pathogenic mechanism was suggested by NGS. Thus, in unresolved cases, complementary methods like genome sequencing and techniques to detect copy number variations (CNVs) (including deletions and duplications), such as MLPA, may be important.

However, challenges remain using NGS, especially in rare syndromes where HPO-based gene prioritization is difficult due to poor description of ocular findings. Precise ophthalmological and genetic assessment remain crucial, but in many syndromic conditions, the ocular phenotype is vaguely described—e.g., “visual impairment” instead of specific findings like coloboma. For example, in *POGZ*-associated White–Sutton syndrome, ocular anomalies or visual impairment are frequently mentioned, yet rarely detailed. This lack of specificity reduces diagnostic sensitivity, especially when the ocular findings are the primary reason for referral. It underscores the need for detailed phenotype documentation and reanalysis of prior negative genetic results if more specific phenotypic description is available.

In 17 cases, no causative variants were found, possibly due to non-genetic causes, non-coding mutations, or undiscovered genotype–phenotype associations [34].

Accurate genetic diagnosis in congenital eye malformations has significant implications for prognosis, therapy, comorbidities and family planning. It also enables access to emerging gene therapies. In several cases, similar clinical phenotypes were linked to different genetic causes, each requiring distinct medical management. For example, *COL4A1* mutations may indicate risk for cerebral hemorrhage, as seen in patient 30, and a timely diagnosis could facilitate personalized risk adapted management. On the other hand, X-linked variants in *NHS* or *BCOR* carry specific familial risks [3,16].

Our study has several limitations. Some clinical data from external reports were incomplete, limiting phenotype accuracy and segregation analysis. Due to evolving sequencing methods and software, in earlier cases newly recognized variants may have been missed. Thus, reported yields likely represent a minimum and support re-testing over time.

Exome-based NGS proved highly effective for diagnosing congenital eye malformations, confirming causative variants in 43% of cases and suspected variants in 15%. Broader panels outperformed phenotype-specific diagnostics and our findings highlight the importance of interdisciplinary cooperation and routine genetic testing. Eight novel pathogenic variants in six genes, rare potential genotype–phenotype associations and candidate genes were identified, advancing understanding of genetic mechanisms in congenital eye malformations.

## 4. Materials and Methods

### 4.1. Patient Selection and Clinical Data Collection

We included patients who presented at MGZ Medical Genetics Center, Munich, for consultation and diagnostics between 2016 and 2021 due to congenital eye malformations with the MAC, ASD, PGC or CC phenotypes. Informed consent was obtained from all participants or their legal guardians and the study was approved by the institutional review board of Ludwig-Maximilians University Munich, Germany (approval number: 19-317). All principles of the Declaration of Helsinki were followed. Clinical data were collected retrospectively, including ophthalmologic findings, family and prenatal history, prior genetic testing and extraocular anomalies.

### 4.2. Genetic Testing Workflow

Blood samples were collected from patients and available relatives. DNA was extracted using standard protocols. Sequencing libraries were prepared using SureSelectXT Target Enrichment System for the Illumina Platform (Agilent Technologies, Santa Clara, CA, USA) for patients 1–26 and the Human Comprehensive Exome + Mitochondrial Panel (Twist Bioscience, San Francisco, CA, USA) for patients 27–40. Sequencing was performed on MiSeq, NextSeq, or NovaSeq platforms (Illumina, San Diego, CA, USA). A stepwise NGS-based diagnostic approach was used. Initial testing (primary diagnostics, PD) targeted small, phenotype-specific virtual panels (<25 kb coding sequence) selected based on clinical presentation. If no pathogenic variants were found, a broader multigene panel (MGP) of 186 eye-related genes was analyzed. For unresolved cases, full exome data were filtered using Human Phenotype Ontology (HPO) terms to prioritize known gene–disease associations (including phenotype-overlapping genes), syndromic genes with ocular involvement and novel candidate genes with ocular expression and loss-of-function (LOF) variants.

### 4.3. Bioinformatic Analysis, Variant Classification and Interpretation

For patients 1–26, sequencing data were analyzed following a standardized in-house pipeline with Burrows–Wheeler Aligner (BWA, Version 0.7.8-r455), SAMtools (Version 1.1), SnpEff (Version 4.0e) and Alamut-Batch (Version 1.3.1, Interactive Biosoftware, Rouen, France). Population (dbSNP, 1000 Genomes, ESP, GnomAD/ExAC) and clinical databases (ClinVar, Emory, MGZ internal, HGMD, Mastermind, COSMIC) supported variant annotation.

For patients 27 to 40, the Varvis software (Version 2.4.0, Limbus Technologies, Rostock, Germany) was used. Variants were classified by a team of genetic and ophthalmological specialists according to the American College of Medical Genetics (ACMG) system [35]. Only variants classified as pathogenic or likely pathogenic (ACMG classes 4 and 5) were reported as causative. Variants of Unknown Significance (VUS, ACMG class 3) were discussed if there was strong phenotype–genotype correlation.

## Figures and Tables

**Table 1:** Clinical details and sequencing results cases with causative variants. het = heterozygote, hom = homozygote, hem = hemizygote, - = not applicable, Known = previously described in the literature/databases, Novel = not previously described in the literature/databases, ø = unknown/not performed, de novo = negative segregation analysis.

Diagnostic Level	Patient ID	Described Ocular Phenotype	Unilateral/Bilateral	Systemic Features	Family History (Phenotype)	Gene	Sequence Variant	Amino Acid Change	Variant Type	ACMG Class	Variant Known/Novel	Segregation/Variant in Family Members?
PD	*ASD/PCG*-8	Congenital glaucoma	Bilateral	-	Negative	*CYP1B1*	c.1168C>A; het/c.1076_1082dup; het	p.Arg390Ser/p.Asp361Glufs*16	Missense/Frameshift	Class 5/Class 5	Known [12]/Novel	ø
PD	*ASD/PCG*-22	ARS, early childhood glaucoma	Bilateral	-	Positive (mother, aunt, grandmother, daughter)	*FOXC1*	Deletion 3’ Probe Exon 1; het	-	Deletion	Class 4	-	ø
PD	*ASD/PCG*-23	Anterior segment dysgenesis (Rieger-anomaly)	Bilateral	Teeth anomalies, excessive umbilical skin	Positive (daughter)	*PITX2*	Deletion of all Probes (Intron 3, Exon 4, Exon 5); het	-	Deletion	Class 4	-	ø
PD	*ASD/PCG*-34	Anterior segment dysgenesis/congenital glaucoma	Bilateral	-	Negative	*FOXC1*	c.247T>C; het	p.Tyr83His	Missense	Class 4	Known [13]	de novo
PD	*CC*-30	Congenital cataract, microcornea	Bilateral	Cerebral hemorrhages	Negative	*COL4A1*	c.2317G>A; het	p.Gly773Arg	Missense	Class 5	Known [14]	de novo
PD	*CC*-33	Congenital cataract, microcornea	Bilateral	-	Negative	*PAX6*	c.113G>A; het	p.Arg38Gln	Missense	Class 4	Known [15]	ø
MGP	*MAC*-9	Microphthalmia, congenital cataract	Bilateral	Radiculomegaly, oligodontia, toe syndactyly, broad nasal tip	Positive (mother, sister)	*BCOR*	c.4038_4039del; het	p.Glu1348Ilefs*26	Frameshift	Class 4	Known [16]	Mother affected
MGP	*MAC*-10	Microphthalmia, congenital cataract	Bilateral	-	Positive (sister)	*NHS*	c.320del; hem	p.Gly107Alafs*89	Frameshift	Class 5	Novel	ø
MGP	*MAC*-11	Iris/retinal/choroidal coloboma	Bilateral	-	Negative	*MAB21L2*	c.58del; het	p.Cys20Valfs*37	Frameshift	Class 4	Novel	ø
MGP	*MAC*-15	Iris/retinal/choroidal coloboma	Bilateral	-	Negative	*MAB21L2*	c.58del; het	p.Cys20Valfs*37	Frameshift	Class 4	Novel	ø
MGP	*MAC*-19	Microphthalmia, glaucoma, extreme hyperopia	Bilateral	-	Positive (sister)	*MFRP*	c.201G>A; het/c.1534T>G; het	p.Trp67*/p.Cys512Gly	Nonsense/Missense	Class 4/Class 4	Known [17]/ Novel	ø
MGP	*MAC*-37	Microphthalmia, congenital cataract	Bilateral	Ureteral stricture, psoriasis, dental duplication	Negative	*NHS*	c.3659dup; hem	p.Asn1220Lysfs*5	Frameshift	Class 4	Novel	ø
MGP	*MAC*-39	Iris/retinal/choroidal/optic disk coloboma	Bilateral	Sandal gap deformity	Negative	*MAB21L2*	c.145G>A; het	p.Glu49Lys	Missense	Class 5	Known [18]	de novo
MGP	*MAC*-40	Microphthalmia	Bilateral	-	Negative	*PRSS56*	c.1066dup; hom	p.Gln356Profs*152	Frameshift	Class 5	Known [19]	ø
MGP	*ASD/PCG*-27	Corneal opacity, iris hypoplasia, optic disk changes, congenital glaucoma	Bilateral	-	Negative	*CYP1B1*	c.1159G>A; het/c.1064_1076del; het	p.Glu387Lys/p.Arg355Hisfs*69	Missense/Frameshift	Class 5/Class 5	Known [20]	ø
MGP	*CC*-4	Congenital cataract	Bilateral	Language-related developmental delay/microcephaly	Positive (brother)	*GCNT2*	c.1148G>A; hom	p.Arg383His	Missense	Class 4	Known [15]	ø
Exome	*ASD/PCG*-20	ARS, high myopia, microcornea, maculopathy, glaucoma	Bilateral	Taurodontism, conspicuous auricle	Positive (sister, brother)	*ADAMTS18*	c.1399G>T; hom	p.Gly467*	Nonsense	Class 5	Novel	Sister affected

**Table 2:** Clinical Variants of Unclear Significance (VUS) and candidate genes. het = heterozygous, hom = homozygous, - = not applicable, Known = previously described in the literature/databases, Novel = not previously described in the literature/databases, ø = unknown/not performed, * = variant published as VUS in the context of diaphragmatic hernia.

Patient ID	Described Ocular Phenotype	Unilateral/Bilateral	Systemic Features	Family History (Phenotype)	Gene	Sequence Variant	Amino Acid Change	Variant Type	ACMG Class	Variant Known/Novel	Segregation/Variant in Family Members?
*MAC*-1	Iris/choroidal coloboma OD, optic disk coloboma OS	Bilateral	Motor development delay	Negative	*POGZ*	c.3271C>T; het	p.His1091Tyr	Missense	Class 3	Novel	ø
*MAC*-5	Iris/choroidal coloboma	Unilateral	Developmental delay	Negative	*POGZ*	c.4086A>C; het	p.Glu1362Asp	Missense	Class 3	Known [21] *	ø
*MAC*-26	Iris/retinal/choroidal coloboma	Bilateral	-	Negative	*FAT1*	c.1025G>C; het	p.Gly342Ala	Missense	Class 3	Novel	ø
*MAC*-28	Microphthalmia, extreme hyperopia, glaucoma	Bilateral	-	Positive (brother)	*MFRP*	c.572T>A; hom	p.Ile191Lys	Missense	Class 3	Novel	Brother affected
*ASD/PCG*-12	Early childhood glaucoma	Unilateral	-	Negative	*COL18A1*	c.2156C>T; het	p.Thr719Met	Missense	Class 3	Novel	ø
*CC*-14	Congenital cataract	Bilateral	-	Positive (mother, sister, 2 daughters, son)	*CRYBB1*	c.588_602dup; het	p.Gln197_Tyr201dup	Intragenic Duplication	Class 3	Novel	Mother, Sister, 2 Daughters, Son affected

## Data Availability

The raw data supporting the conclusions of this article will be made available by the authors on request.
